# Repetitive Transcranial Magnetic Stimulation for Adolescent Major Depressive Disorder: A Focus on Neurodevelopment

**DOI:** 10.3389/fpsyt.2021.642847

**Published:** 2021-04-13

**Authors:** Lindsay M. Oberman, Megan Hynd, Dylan M. Nielson, Kenneth E. Towbin, Sarah H. Lisanby, Argyris Stringaris

**Affiliations:** National Institute of Mental Health, Bethesda, MD, United States

**Keywords:** depression, rTMS, adolescence, neurodevelopment, individualized targeting

## Abstract

Adolescent depression is a potentially lethal condition and a leading cause of disability for this age group. There is an urgent need for novel efficacious treatments since half of adolescents with depression fail to respond to current therapies and up to 70% of those who respond will relapse within 5 years. Repetitive transcranial magnetic stimulation (rTMS) has emerged as a promising treatment for major depressive disorder (MDD) in adults who do not respond to pharmacological or behavioral interventions. In contrast, rTMS has not demonstrated the same degree of efficacy in adolescent MDD. We argue that this is due, in part, to conceptual and methodological shortcomings in the existing literature. In our review, we first provide a neurodevelopmentally focused overview of adolescent depression. We then summarize the rTMS literature in adult and adolescent MDD focusing on both the putative mechanisms of action and neurodevelopmental factors that may influence efficacy in adolescents. We then identify limitations in the existing adolescent MDD rTMS literature and propose specific parameters and approaches that may be used to optimize efficacy in this uniquely vulnerable age group. Specifically, we suggest ways in which future studies reduce clinical and neural heterogeneity, optimize neuronavigation by drawing from functional brain imaging, apply current knowledge of rTMS parameters and neurodevelopment, and employ an experimental therapeutics platform to identify neural targets and biomarkers for response. We conclude that rTMS is worthy of further investigation. Furthermore, we suggest that following these recommendations in future studies will offer a more rigorous test of rTMS as an effective treatment for adolescent depression.

## Introduction

Adolescent depression is a leading cause of disability, yet its treatment remains unsatisfactory. Thus, there exists an urgent need for novel, neurodevelopmentally-informed, targeted therapeutics. Repetitive Transcranial magnetic stimulation (rTMS) has emerged as a promising treatment modality for adult major depressive disorder (MDD). rTMS is a non-invasive method to modulate brain network functioning through the application of pulsed magnetic fields (1). Several reviews concluded that rTMS could be a potentially safe and effective treatment for adolescent depression (2–6), however, empirical studies yield mixed results.

Indeed, the sole large-scale (*n* = 103) randomized controlled trial (RCT), an industry-sponsored effort to extend FDA clearance for rTMS to depression in adolescents, was negative (7), that is, did not show a difference between rTMS and sham control.

In light of this trial, this review focuses on how neurodevelopment creates challenges for development of rTMS protocols in this population and provides specific recommendations to overcome these complexities. We highlight the developmental pathophysiology underlying symptoms of adolescent depression and relate these to the putative mechanisms of action of rTMS. We propose that future studies employ experimental therapeutics approaches to identify predictive biomarkers of response and to develop individualized, neurodevelopmentally informed rTMS targets.

Relevant studies were ascertained via a literature search of PubMed and Google Scholar. The search was limited to English-language peer-reviewed articles. The search terms used were: “adolescent depression” and “TMS” or “rTMS.” Also, to ensure that we accurately represented the full extent of the literature, we examined previously published review articles. We excluded studies that applied single-pulse TMS, paired-pulse TMS, or other non-therapeutic TMS protocols, trials. We also excluded studies that evaluated depressive symptoms in adolescents with other primary clinical conditions [e.g., Tourette syndrome or autism spectrum disorder (8, 9)].

## A Neurodevelopmental Overview of Adolescent Depression

The World Health Report suggests that depression is the leading cause of disability worldwide, affecting over 264 million people (10, 11). The prevalence of moderate-to-severe depressive symptoms in youth between the ages of 12 and 17 is estimated to be 5.7% (12) with a cumulative prevalence of around 10% by age 16 (13). Moreover, depressed adolescents are about 30 times more likely to commit suicide compared to their non-depressed counterparts (14). Suicide is one of the leading causes of death in adolescents in the US and adolescence is also the time of peak incidence of suicidal behaviors and suicidal ideation (15). Despite the substantial individual and societal impact associated with depression in youth, treatment options are limited (16, 17). Traditional treatment methods include psychopharmacology (e.g., serotonin reuptake inhibitors) and behavioral therapy [e.g., cognitive behavioral therapy (CBT)]. The Treatment of Adolescent Depression Study (TADS), however, found that only 37% of patients experienced full remission of symptoms after 12 weeks of these first-line treatments (18). Furthermore, even with the combination of two evidence-based treatment modalities, at least a third of youths treated for depression do not respond, 20–37% only have a partial response, and 40–70% experience a relapse or recurrence (19–21). Thus, there remains a significant need for the development of new treatments.

In addition to the morbidity and mortality, there is significant financial burden of adolescent depression including: costs of health care use, productivity lost, and time off of work for caregivers. Estimates of direct costs of adolescent depression amount to ~$2,900 additional dollars per year. This does not consider the indirect costs of reduced/lost productivity, which in adult depression is estimated as high as $12,000 per year (22). Given this financial burden, the cost-efficiency of treatment options is also a consideration. Pharmacotherapy, estimated at $100 per month is the least expensive therapy, followed by psychotherapy, estimated at $100–$150 *per session*. Both pharmacotherapy and psychotherapy are significantly less expensive than rTMS for a given depressive episode. However, one industry-sponsored study of individuals who failed a single course of antidepressants, applied simulation modeling to compare costs of rTMS therapy to multiple serial medication trials and suggested that rTMS may cost less in over the course of the patients' lifetime (23).

Adolescence is not only a time when the incidence of depression increases (24), but also a period of substantial social, emotional, and biological development. These developmental changes may contribute to risk factors and mechanisms underlying adolescent depression (25). Synapses in the adolescent brain are highly dynamic; new synapses are formed and others eliminated at higher rates than seen in adults (26, 27). Proposed developmental models of the increased risk of psychopathology in adolescence point to a mismatch in the growth of brain networks supporting emotional reactivity and regulation. Compared to brain networks subserving emotion regulation, those pertaining to emotional reactivity develop more rapidly (28, 29). The prefrontal cortex (PFC) is a key node in the emotion regulation network underlying complex cognitive tasks such as inhibition, working memory, cognitive control, and attention. The PFC undergoes age-dependent functional changes well into late adolescence and early adulthood (30–32). Structural neuroimaging shows decreases in total gray matter PFC volume starting at around 11–12 years old (33, 34). This decrease is thought to be associated with synaptic pruning (27). In contrast, imaging metrics of myelination, axon density and white matter volume in frontal regions, show relatively linear increases across adolescence (35–39). It is postulated that an imbalance of the immature PFC and the more mature frontal subcortical systems regulating emotional reactivity might lead to a predominance of “bottom-up” emotional reactivity (40–44).

On a molecular level, these adolescent neurodevelopmental changes are thought to result from fluctuations in neurotransmitter concentration and receptor expression. Notably, fluctuations in neurotransmitter systems, such as GABA (45, 46), NMDA (47), and dopamine (48, 49) have profound impact on neural signaling in regions pertaining to emotion regulation. GABAergic and glutamatergic systems in the PFC and cingulate cortices have a direct impact on the excitability and plasticity in regions subserving emotion regulation (50). Fluctuating dopamine levels and emergence of dopamine receptor-mediated facilitation of NMDA (glutamate) receptor transmission and GABAergic interneuron excitability both have been proposed as a mechanism of increased sensitivity to rewards, novelty, or other salient stimuli (51–53). These cellular and molecular changes in adolescence lead to imbalances in excitation and inhibition, changes in cortical plasticity and connectivity, and less effective transferring of information between critical emotion processing brain regions (50, 54).

The pathophysiology that underlies adolescent depression may differ from that in adults (54). Studies in clinical samples and animal models suggest that these aberrant maturational processes contribute to adolescent depression (55). In one magnetic resonance spectroscopy (MRS) study, adolescents with depression showed decreased levels of GABA in the anterior cingulate cortex (ACC), as compared to healthy adolescents. Furthermore, this difference was specifically related to anhedonic symptoms (56). Another study found that symptom severity in both adults and adolescents with depression correlated with GABA and glutamate + glutamine (Glx) concentrations in the PFC (57, 58).

Neurodevelopmental changes and pathophysiology need to be considered when designing trials of novel targeted therapeutics, such as rTMS. In the next section we review the literature on rTMS. We suggest that future rTMS protocols may benefit from applying neurodevelopmentally-informed approaches of modulating aberrant brain networks and neurotransmitters to treat adolescent depression.

## Repetitive Transcranial Magnetic Stimulation in Adult and Adolescent Major Depressive Disorder

TMS is a non-invasive neuromodulation technique that is increasingly utilized in clinics and laboratories world-wide to study and treat a range of neurological and psychiatric disorders. In research, TMS can be applied in single pulses to depolarize a small population of neurons in a targeted brain region. Single-pulse TMS can be used to measure cortical excitability, study central motor conduction time, or the cortical silent period (a measure of intracortical inhibition), or map effective connectivity between the stimulated region and other brain regions (59). TMS can also be applied in pairs of pulses (i.e., paired-pulse stimulation); two pulses are presented in rapid succession to study intracortical inhibition and facilitation (60, 61).

During rTMS, trains of regularly repeating TMS pulses are applied at various stimulation frequencies (e.g., 1, 5, 10 Hz) and patterns [e.g., Theta Burst Stimulation (TBS) (62) or Quadrapulse Stimulation (QPS) (63)]. Compared to paired-pulse or single-pulse stimulation protocols, rTMS pulses temporally summate to produce longer lasting changes in neural activity (64). Stimulation frequencies 1 Hz or lower are thought to produce local cortical inhibition while those 5 Hz or higher are thought to generate local cortical excitation (64, 65). There are also specific patterned forms of rTMS including intermittent theta burst stimulation (iTBS) and continuous theta burst stimulation (cTBS). iTBS and cTBS protocols lead to long-lasting facilitation and suppression of cortical excitability, respectively. Compared to 30 min or more for the standard 10 Hz rTMS procedures, a single session of cTBS and iTBS takes ~40 s and 3 min, respectively.

At a system-level, rTMS modulates excitability in targeted regions of stimulation (66–69) and exerts broader effects across networks connected to those regions (70–74). Thus, in adolescents with depression, rTMS applied to PFC could mitigate some regional prefrontal pathophysiology and aberrant functional connectivity between PFC and the limbic system. Furthermore, if successful, modulating these systems during adolescence, a critical period of PFC maturation, could potentially generate longer term clinical benefits than seen in adults. However, the degree and direction of neurophysiological effect of rTMS are influenced by the state of excitability of the targeted cortical region and the degree of functional connectivity across the targeted network (75, 76). In addition, as noted above, there is considerable inter-individual variability at the symptom and pathophysiological level. Thus, it is important to characterize the current brain state in terms of local cortical excitability and network connectivity in order to determine the optimal treatment protocol for a given individual. As will be described below, identifying the optimal target and protocol for a given individual remains theoretical due to the complex etiology of adolescent depression.

### Safety and Mechanisms of Action of rTMS in Adult and Adolescent MDD

The safety of TMS in clinical practice and research has been evaluated through multiple meta-analyses (77–80). Safety guidelines have also been disseminated by the International Federation of Clinical Neurophysiology (81–83). Widespread application of several TMS protocols, across diverse populations and devices, show a low incidence of Adverse Events (AEs) (84). This safety record led to FDA clearance of rTMS for the treatment of adult MDD and adult obsessive-compulsive disorder (OCD) in 2008 and 2018, respectively.

Initial rTMS trials in depression were based on theory that the clinical symptoms might arise from an imbalance between PFC hypometabolism and the limbic system (85). Early studies aimed to increase excitability in regions of PFC that were thought to influence regulation of the limbic system (66, 86–88). Subsequent blood oxygen-level dependent (BOLD) functional magnetic resonance imaging (fMRI) studies suggest that rTMS modulates both activity and connectivity of the targeted region and related networks (74, 89–94). Of note, the cellular and molecular mechanism of action of rTMS is still under investigation (95). At a system level, the Human Connectome Project (96, 97) has led to development of resting state functional connectivity (RSFC) signatures that are being proposed as individualized rTMS targets (98–101). It has also been suggested that some of the changes seen on fMRI following rTMS may result from modulations in GABA and glutamatergic systems (2, 57, 102–106).

As compared to the adult literature, the data on the safety of rTMS in adolescent depression are lacking. However, the data that exist suggest a similar safety profile in older children and adolescents as compared to adults. Allen et al. (107) conducted a systematic review of TMS safety in pediatric populations (including healthy volunteers and youth with neurological and/or psychiatric disorders) in 2017. Forty-two single-pulse and/or paired-pulse TMS studies (*n* = 1,205) and 26 rTMS studies (*n* = 360) were reviewed. Adverse event rates ranged from 3.4 to 10.11% and varied based on the patient population being studied, the form of TMS being applied, and the number of sessions applied. Those with known neurological disorders or those receiving epileptogenic medications for psychiatric disorders were more at risk of adverse events. Similarly, adverse events were more common in high-frequency and/or high intensity rTMS protocols and protocols that involved a higher number of sessions (107). In 2020, Zewdie et al. (108), who run a pediatric brain stimulation clinical research program, published a report on their experience with the safety and tolerability of TMS in a cohort of 384 youth (108). The individuals in this report included healthy volunteers (*n* = 118), patients with perinatal stroke (*n* = 101), patients with mild traumatic brain injury (*n* = 121), and patients with neuropsychiatric disorders (*n* = 37). They report no serious adverse events and excellent tolerability despite over a hundred patients who were at greater risk for seizure due to a neurological condition. As with previous reports, Zewdie et al. note that the most common side-effects were transient headache and neck pain. The authors conclude that standard TMS paradigms, including single pulse, paired-pulse, and rTMS, should be considered minimum risk and provide a safety and tolerability evaluation form for use in this population.

In 2015, Krishnan et al. conducted a safety review involving 35 studies (*n* = 322) focused on the use of rTMS in children and adolescents with range of conditions (109). Fifteen studies reported no adverse events or that the treatment was “well-tolerated,” without specifying adverse events. The most common adverse events reported were headache (11.5% of patients) and scalp discomfort (2.5%). A third pediatric TMS safety study was conducted by Hong and colleagues in 2015 and focused specifically on the safety and tolerability a novel rTMS paradigm, namely TBS (110). This retrospective analysis (*n* = 76) reported adverse events in 10.5% of TBS sessions including: headache, arm/hand/other pain, numbness/tingling, and weakness (110). The rate and severity of adverse events reported in this study did not differ between those that received TBS and a comparator group of 89 youth who received single- and paired-pulse TMS. Similar rates were also reported for active and sham (placebo) TBS (110).

More serious adverse events in pediatric studies have been rare (occurring in ~1–2% of participants). Two cases of syncope in children with pediatric stroke and six cases of seizures (four in adolescents with depression, one in an adolescent with migraines, and one in an adolescent with schizophrenia) have been reported (111–116). Factors that could have increased the risk of these serious adverse events include: concomitant medication use (in four of the cases), alcohol withdrawal (in one case), and clinical disorders associated with increased risk of syncope and seizure (i.e., pediatric stroke and migraine, respectively). More details about these case reports can be found in Table 1.

**Table 1:** Case reports of TMS induced seizures in adolescents.

**Publication**	**Patient status**	**Age, gender**	**TMS protocol**	**Intensity**	**Location**	**Stimulator model/Coil type**	**Seizure description**
Hu et al. (111) Journal of International Medical Research	MDD	15 (F)	10 Hz rTMS	100% RMT	L- Prefrontal Lobe	Magstim Figure 8 Coil	Generalized tonic-clonic seizure, started within minutes of 1st treatment
Chiramberro et al. (112) Brain Stimulation	MDD	16 (F)	10 Hz rTMS	Not reported	L-DLPFC	Magstim Figure 8 Coil	Generalized tonic-clonic seizure induced 20 min into 40 trains on the 12th day of stimulation
Cullen et al. (113) Journal of Child and Adolescent Psychopharmacology	MDD	17 (F)	18 Hz Deep TMS	120% RMT	L- Motor cortex	H1 Coil	Generalized tonic-clonic seizure induced on the 48th train of the 8th day of treatment
Wang et al. (114) Brain Stimulation	Migraine	16 (F)	10 Hz rTMS	110% RMT	L- Motor cortex	Magstim Rapid Figure of 8 Coil	Generlized tonic-clonic seizure induced 10 seconds into the 3rd train of the 1st session
Purushotham et al. (115) Brain Stimulation	Schizophrenia	15 (F)	iTBS	80% AMT	L-Motor cortex	Magstim Rapid Figure of 8 Coil	Generalized tonic-clonic seizure induced 30 seconds into the 1st session
Kallel and Brunelin (116) Journal of ECT	MDD	18 (F)	20 Hz rTMS	110% RMT	L-DLPFC	MagPro X30 Figure of 8 Coil	Generlized tonic-clonic seizure induced on the 26th train of the 3rd session on the 2nd day of stimulation

*MDD, Major Depressive Disorder; rTMS, repetitive transcranial magnetic stimulation; iTBS, intermittent theta-burst stimulation; RMT, resting motor threshold; AMT, active motor threshold; L-DLPFC, left dorsolateral prefrontal cortex*.

The most recent International Federation of Clinical Neurophysiology TMS safety guidelines indicate that the extant pediatric literature “provide reassurance regarding the safety of these techniques” in pediatric populations (82). However, as noted above, this “reassurance” is based on far less data than in the adult literature. Furthermore, the neurodevelopmental processes ongoing in children and adolescence, compounded by the pathophysiological processes affecting those with neuropsychiatric and neurodevelopmental disorders require consideration (117, 118). As it relates to adolescent depression, the aforementioned neurotransmitter fluctuations and potentially aberrant functional connectivity may result in an altered neurophysiological state (as compared to the adult brain). Thus, evaluating and adjusting for the physiological state of the brain may both reduce the risk of adverse events as well as potentially increase the intended effects.

### Efficacy of rTMS in Adult and Adolescent MDD

#### Efficacy of rTMS in Adult MDD

In addition to publishing safety guidelines, the International Federation of Clinical Neurophysiology has also published a series of evidence-based guidelines on the therapeutic use of rTMS (119, 120). To develop these guidelines, experts in the field evaluated the level of evidence of rTMS efficacy for a number of indications. Consistent with the FDA label and based on a number of large-scale clinical trials (121–123), high-frequency (10 Hz) rTMS to the left dorsolateral prefrontal cortex (DLPFC) achieved a “Level A” (definite efficacy) for adult MDD. Since its initial FDA clearance, in 2008, multiple TMS devices and protocols have also received clearance for adult MDD. Consensus guidelines have also been established by the National Network of Depression Centers and the American Psychiatric Association Council on Research (124). Multiple meta-analyses of thousands of individuals have concluded that rTMS is safe, tolerable, and leads to a reduction in depressive symptoms in otherwise treatment resistant adult patients with MDD. Although the safety and tolerability of rTMS is consistent across trials, effect sizes vary greatly based on a number of factors including: rTMS parameters (e.g., intensity, location, and stimulation protocol) and interindividual factors such as brain size, shape, and neurophysiological state.

Intensity of stimulation is typically set in relation to the individual's motor threshold (MT). The MT is the stimulator output that is required to produce a contraction of the thumb or fingers half the time when applied to the primary motor cortex “hotspot.” MT is used as a proxy of the intensity of stimulation necessary to activate other regions of cortex. Intensity of rTMS typically ranges from 80 to 120% of MT. At these intensity levels, current models indicate that standard coils induce an electrical field that can reach 2–3 cm from the scalp (125). Consistent with the FDA label for adult MDD, the DLPFC target is often approximated from measurements on the scalp (126). However, some studies have also used structural or functional MRI combined with a frameless stereotaxic neuronavigation system to target specific regions of interest (65, 127). Targeting rTMS based on fMRI and/or diffusion tensor imaging (DTI) mapping of an individual's brain network (128, 129) tends to result in larger effect sizes as compared to scalp-based approaches (127, 130, 131). Typical stimulation frequencies vary from 1 to 20 Hz. The most common frequency used for adult MDD is 10-Hz. rTMS applied to the DLPFC at 10 Hz frequency per the FDA label is associated with an average of 30% response rate, compared with 10.4% with sham (placebo) rTMS, with an effect size of 0.55 (132–134) and a pooled odds ratio of response or remission of 3.3 (132). Furthermore, according to a recent meta-analysis, 66.5% of individuals who respond to the initial rTMS course have sustained response after 3 months, with responder rates decreasing to 52.9% after 6 months, and 46.3% of individuals still maintaining response 1 year after the initial treatment course (135).

Several approaches have been taken to address relapse and recurrence after initial rTMS response. These include various maintenance rTMS schedules as well as additional courses of rTMS treatments during periods of relapse. Though a number of studies have explored various maintenance rTMS schedules [see (136)], protocols vary across studies and clinics. Proposed maintenance regimen involve an initial tapering of sessions over the course of 3-4 weeks from five sessions per week down to one session every week and eventually one session every 2 or 3 weeks for many months to several years depending on the individual [see (137)]. Though this regimen may provide optimal protection against relapse, there may be alternative options for maintenance that are less burdensome including introduction and/or modifications of antidepressant medication or psychotherapy.

The effects of rTMS are not simply a matter of the stimulation parameters, but also how the stimulation is received and processed in the brain. The specific degree and location of stimulation of the targeted brain region depends upon the individually unique structural and functional architecture of each individual's brain. Given the putative mechanism of action of rTMS is the modulation of functional networks and the known interindividual structural and functional heterogeneity of these networks, the efficacy of rTMS depends upon the accurate targeting of the network of interest in that individual. Furthermore, although high-frequency and iTBS protocols typically lead to increased excitability of the targeted region, recent studies report considerable inter-individual and intra-individual (state-dependent) variability in cortical response, especially outside of the primary motor cortex (138, 139). Thus, efficacy can be optimized through careful characterization of both the functional network architecture and neurophysiological state.

#### Efficacy of rTMS in Adolescent MDD

Most of what is known about the effects of rTMS on the brain are based on adult studies. Less than 1,000 children and adolescents are represented in the published rTMS literature. Without FDA clearance, commercial marketing of rTMS for any pediatric indications is prohibited. While adolescents and children may be studied under an approved research protocol, any other use of TMS in individuals under age 21 is “off-label” in the United States.

The first published review on rTMS in adolescent depression (6) was based on two case series (140, 141) and one open-label trial (142) (*n* = 14). With so few data, no meaningful conclusions could be made about efficacy. The authors suggested that the optimal stimulation parameters for adolescents might differ from those in adults. A subsequent review (3) included another open-label trial (143), a case report of an induced seizure in an adolescent (111), an open-label trial where depressive symptoms were evaluated in adolescents with Tourette syndrome (8), and a secondary analysis on the previous open-label trial. Despite the expanded number of publications, the rTMS literature was comprised of only 22 (inclusive of the one seizure report) adolescents with a primary diagnosis of MDD. A third review, a decade after the first (4), added three additional case reports (112, 113, 144), a case report of an individual with autism spectrum disorder and co-morbid depression who received rTMS for depressive symptoms (9), a case series (106), an open-label study (58), and three secondary analyses on previous datasets (145–147). By 2017, despite more publications, <50 adolescents with primary MDD had received rTMS and no studies included a placebo control group. The next review (2), in 2019, included two additional open-label trials (148, 149), more than doubling the previous total number of depressed adolescents treated with rTMS. This review focused on the effects of rTMS on GABAergic and glutamatergic neurotransmission and concluded by calling for larger, neurodevelopmentally-informed studies. The most recent review (5), included one additional case series (150), one new retrospective analysis of clinical data (151), and two more secondary analyses of existing datasets (152, 153) (*n* = ~150). The authors (5) conclude by highlighting flawed study designs, calling for sham-controlled RCTs to properly assess the efficacy of rTMS.

Since the publication of the most recent review article, there is now one sham-controlled RCT of rTMS in adolescents with MDD (7) and two additional retrospective analyses of clinical data (154, 155). With the addition of these new data, the adolescent depression rTMS literature now encompasses 20 publications (Table 2) representing 12 unique datasets (*n* = ~280 adolescent participants with MDD). Despite the calls for neurodevelopmentally-informed trials, the parameters have mimicked those in the adult studies. Intensity of stimulation has ranged from 80 to 120% of motor threshold. Protocols have largely followed the FDA clearance (for adults) applying 10 Hz unilaterally to the left DLPFC. Two studies took a different path. One (151) compared 1 Hz, unilateral right DLPFC stimulation with a bilateral (left followed by right) stimulation and another applied bilateral TBS stimulation (148). As for sample characteristics, studies have taken a conservative approach, enrolling relatively older adolescents (average age of 17.15), small sample sizes (7/12 datasets with sample sizes n ≤ 10), and open-label designs or active stimulation case reports/retrospective analyses of clinical data (11/12 datasets). The sole exception is the large-scale RCT that enrolled 103 adolescents ~18% of whom were age 12–14 years old (7).

**Table 2:** Previous literature on TMS for adolescent depression.

**Publication**	***n***	**Age in years (Mean, Range)**	**Gender**	**Protocol**	**Number of sessions, Frequency of sessions**	**Depression outcome measure**	**Subject medications**	**Estimated effect size**	**Reported effects for depression outcome measure**	**Side-effects/Adverse events**
Walter et al. (141) Journal of Child and Adolescent Psychopharmacology	*n =* 3	Ages: 16, 17, and 17	3 males	10 Hz rTMS, 90–110% RMT, over LDLPFC	10 treatment sessions over 2 weeks	HAM-D & BDI	None	Hedges's g_av_ = 1.53, Glass's Δ_pre_ = 3.37	Improvement of HAM-D from 28 (baseline) to 8 (week 4) for one participant Improvement of HAM-D from 34 (baseline) to 12 (week 4) for one participant No improvement for one participant	Adverse effects in only one patient—tension headache in two sessions
Loo et al. (140) Australasian Psychiatry	*n =* 2	Ages: 16, 16	Both female	10 Hz rTMS at 110% RMT; 40 trains of 5 second duration, 25 second ITI	29–36 treatment sessions over 6–11 weeks	MADRS, CGIS, BDI, Centre for Epidemiological Studies—Depression-Child Scale	*n =* 1: “psychotropic medication,” *n =* 1: venlafaxine and methylphenidate	n/a	“Both subjects improved to a clinically significant degree with rTMS treatment	No adverse effects
Bloch et al. (142) The Journal of ECT ^*^Mayer et al. (156)	*n =* 9	M = 17.3 Range = 16–18	2 males, 7 females	10 Hz rTMS at 80% RMT over LDLPFC (5 cm targeted); 20 trains, 2 s per train	20 treatment sessions over 2 weeks	CDRS, Screen for Child Anxiety-Related Disorders, Suicidal Ideation Questionnaire CGIS, Cambridge Neuropsychological Test Automated Battery	Not reported	Hedges's g_av_ = 1.50, Glass's Δ_pre_ = 2.63	Response rate of 33%	No adverse effects reported
Wall et al. (143) The Journal of Clinical Psychiatry ^*^Croarkin et al. (147) ^*^Wall et al. (146) ^*^Croarkin et al. (153) ^*^Somnez et al. (157)	*n =* 7	M = 16.5 Range =14.6–17.8	1 male, 6 females	10 Hz rTMS at 120% RMT over LDLPFC (5 cm targeted); train duration of 4 s, 26 s ITI, total 3,000 pulses	30 treatment sessions over 6–8 weeks	CDRS-R, QIDS-A17, CGI-S, Suicide Severity Scale	Not reported	Hedges's g_av_ = 4.51, Glass's Δ_pre_ = 5.05	CDRS-R scores improved from treatment 10 (mean = 50.9, SD = 12, *P* < 0.02) to treatment 30 (mean = 32.6, SD = 7.3, *P* < 0.0001), and at 6-month follow-up (mean = 32.7, SD = 3.8, *P* < 0.0001)	Scalp discomfort in 3 out of 8 participants
Yang et al. (106) The Journal of ECT	*n =* 6	M = 18.7Range = 15–21	2 males, 4 females	10 Hz rTMS at 120% RMT over LDLPFC (structural-MRI targeted); 4 s trains, ITI 26 s, 75 trains, 3,000 pulses	15 treatment sessions over 3 weeks	HAM-D, BDI	Not reported	Hedges's g_av_ = 2.63, Glass's Δ_pre_ = 3.18	Response rate of 66% Responders had an 11% increase in glutamate levels from baseline	No adverse events reported
Segev et al. (144) The Journal of ECT	*n =* 1	17	1 male	10 Hz rTMS, 100% RMT over LDLPFC (5 cm targeted), 42 trains of 4 s with an ITI of 30 s, 1,680 pulses per treatment	20 treatment sessions over 4 weeks	BDI-II, SIQ, Childhood Anxiety Related Disorder Questionnaire	Not reported	n/a	“…significant clinical improvement was demonstrated in anxiety symptoms and not in clinical measures of depression”	Headache, scalp pain, and scalp burning
Croarkin et al. (58) Psychiatry Research: Neuroimaging ^*^Wall et al. (145) ^*^Croarkin et al. (153) ^*^Sonmez et al. (152)	*n =* 10	M = 15.4 Range = 13.9–17.4	6 males, 4 females	10 Hz rTMS at 120% RMT over LDLPFC (structural-MRI targeted); train duration of 4 s, 26 s ITI, total 3,000 pulses	30 treatment sessions over 6–8 weeks	CDRS-R, QIDS-A17-SR, CGI-S	Not reported	Hedges's g_av_ = 1.89, Glass's Δ_pre_ = 2.57	CDRS-R total score at baseline was 62.9 (SD = 8.2), total score at posttreatment was 41.8 (SD = 13.2), total score at 6-month follow up was 34.2 (SD = 15.3) Also reported, “…throughout the 6-month follow-up period, we estimated that a 1–scale unit increase (or decrease) in the CDRS-R total score (depression severity) was related to a mean decrease (or increase) in each Gln/Glu ratio”	Scalp discomfort, headaches, dizziness, neck stiffness, eye twitching, nausea, musculoskeletal discomfort
MacMaster et al. (149) Frontiers in Psychiatry	*n =* 32	M = 17.57 Range = 13–21	17 males, 15 females	10 Hz rTMS at 120% over LDLPFC (structural-MRI targeted); 4 s trains, ITI 26 s, 75 trains, 3,000 pulses	15 treatment sessions over 3 weeks	HAM-D	Not reported	Hedges's gav = 1.82, Glass's Δpre = 1.71	Response rate of 56% Remission rate of 44%	Limiting headaches and mild neck pain
Zhang et al., pooled analysis ^*^Zhang et al. (150) Brain Stimulation ^*^Zhang et al. (155) Journal of Affective Disorders ^*^Zhang et al. (154) Journal of ECT	*n =* 70 2 weeks *n =* 23 4 weeks	M = 14.86 Range = 10–17	26 males, 44 females	10 Hz rTMS at 120% MT over LDLPFC (5 cm targeted); 80 trains, 30 pulses per train, 12s ITI, 2,400 pulses or 1 Hz rTMS at 120% MT over RDLPFC (5 cm targeted); 2 trains, 700 pulses per train, 1 s ITI, 1,400 pulses	20 treatment sessions over 4 weeks	HAM-D & HAMA	Sertraline, venlafaxine, duloxetine, mirtazapine *n =* 1: agomelatine, bupropion, deanxit. and clomipramine	Hedges's g_av_ 2 weeks = 1.65, Glass's Δ_pre_ 2 weeks = 1.40 Hedges's g_av_ 4 weeks = 2.85, Glass's Δ_pre_ 4 weeks = 1.98	2-week response rate of 50% 2-week remission rate of 54.3% 4-week response rate of 100% 4-week remission rate of 91.3%	No serious adverse events reported. Limited headaches or musculoskeletal discomfort
Rosenich et al. (151) Early Intervention in Psychiatry	*n =* 15	M = 20.69 Range = 17–25	7 males, 8 females	Unilateral treatment = continuous 1 Hz rTMS over RDLPFC for 15 min (*n =* 2) or 30 min (*n =* 9); Bilateral treatment (*n =* 4) = intermittent 10 Hz rTMS 5 s intervals, 25 s ITI for 1,500 pulses over LDLPFC and followed by 15 min of 1 Hz unilateral treatment for 900 pulses over RDLPFC (all 6 cm targeted). All stimulation at 110% RMT	18 treatment sessions over 6 weeks	HAM-D, MADRS, and Zung Self Rating Depression Scale	Not reported	Hedges's g_av_ = 1.24, Glass's Δ_pre_ = 1.41	Partial response rate of 86.7% Response rate of 40% Remission rate of 13%	No serious adverse events, only mild headache, fatigue, and localized discomfort
Dhami et al. (148) Journal of Affective Disorders	*n =* 20	M = 20.9Range = 16–24	10 males, 10 females	Bilateral theta burst stimulation: iTBS on LDLPFC and cTBS on RDLPFC at 80% RMT (structural-MRI targeted)	10 treatment sessions over 2 weeks	HRSD-17, BDI-II, Q-LES-Q, CDRS-R	Not reported	Hedges's gav = 2.21, Glass's Δpre = 3.07	Response rate of 20% Remission rate of 10%	Headache, scalp pain, chest tightness, anxiety, nausea, gastrointestinal symptoms, nasopharyngitis, restlessness, general discomfort
Croarkin et al. (7) Neuropsychopharmacology*Active arm*	*n =* 48	M = 17.6Range = 12–21	18 males, 30 females	10 Hz rTMS at 120% over LDLPFC (5 cm targeted); 4 strains, 26 s ITI, 75 trains, total 3,000 pulses	30 treatment sessions over 6 weeks	HAM-D, MADRS, CRS-R, QIDS-A-SR, CGI-S	zaleplon, zolpidem, zopiclone, or lorazepam	Hedges's g_av_ = 1.27, Glass's Δ_pre_ = 1.86	Response rate of 41.7%; remission rate of 29.2%	Four serious adverse events reported, all having to do with suicidal ideation or worsening depressive symptoms determined unrelated to rTMS treatment
Croarkin et al. (7) Neuropsychopharmacology*Sham arm*	*n =* 55	M = 17.4Range = 12–21	18 males, 37 females	Sham	30 treatment sessions over 6 weeks	HAM-D, MADRS, CRS-R, QIDS-A-SR, CGI-S	zaleplon, zolpidem, zopiclone, or lorazepam	Hedges's g_av_ = 1.15, Glass's Δ_pre_ = 1.53	Response rate of 36.4%; remission rate of 29.0%	One serious adverse event of suicidal ideation definitely unrelated to rTMS treatment
Croarkin et al. (7) Neuropsychopharmacology*Active* vs. *Sham*	*n =* 103 (54 active)	M = 17.35Range = 12–21	36 males, 67 females	10 Hz rTMS at 120% over LDLPFC (5 cm targeted); 4 s trains, 26 s ITI, 75 trains, total 3,000 pulses	30 treatment sessions over 6 weeks	HAM-D, MADRS, CRS-R, QIDS-A-SR, CGI-S	zaleplon, zolpidem, zopiclone, or lorazepam	Hedges's g_s_ = 0.10,	“There were no statistically significant differences in clinical outcomes between the active TMS and sham TMS groups”	Five serious adverse events reported, all having to do with suicidal ideation or worsening depressive symptoms determined unrelated to rTMS treatment

*rTMS, repetitive transcranial magnetic stimulation; iTBS, intermittent theta burst stimulation; cTBS, continuous theta burst stimulation; RMT, resting motor threshold; MT, motor threshold; ITI, intertrain interval; LDLPFC, left dorsolateral prefrontal cortex; RDLPFC, right dorsolateral prefrontal cortex; HAM-D/HRSD, Hamilton depression rating scale; HAM-A, Hamilton anxiety rating scale; BDI, Beck depression inventory; MADRS, Montgomery-Asberg depression rating scale; SIQ, suicidal ideation questionnaire; CGIS, clinical global impressions scale; CDRS-R, depression rating scale for children revised; QIDS, Quick Inventory of Depressive Symptomatology; Q-LES-Q, quality of life enjoyment and satisfaction questionnaire; SSRI, selective serotonin reuptake inhibitor*.

* *Follow up studies/post-hoc analysis using the same participant data*.

Acknowledging the limitations of the method, open-label trials and case series/retrospective analyses of clinical data were generally positive, with large effect sizes open-label trials: Hedges's g_av_ = 2.39 and Glass's Δ_pre_ = 3.01; case reports/retrospective analyses: Hedges's g_av_ = 2.06 and Glass's Δ_pre_ = 3.48, [see (158) for explanation of effect size measures]. Average reduction in depressive symptoms for the open-label trials and case series/retrospective analyses equals 40 and 51%, respectively (with study averages ranging from 23 to 71%). Open-label trials and case series/retrospective analyses inherently inflate effect sizes and are influenced by regression to the mean, investigator biases, and confounding the effect of the active treatment with placebo effects (159). The only large-scale RCT of rTMS in adolescent depression yielded an effect size near zero (Hedges's g = 0.10). The active and sham rTMS groups showed comparable response (41.7% for the active and 36.4% for the sham group) and remission rates (29.2% for the active and 29.0% for the sham group) (7).

Sham TMS coils have been used in rTMS trials as an analog to pill placebo in drug trials. Sham coils do not apply a magnetic field but mimic the auditory, visual, and (in some cases) sensory experience of rTMS (160). The largest placebo effects have been seen in trials employing “physical placebo interventions” (e.g., a sham TMS coil) with subjective patient-related outcomes (e.g., self or parent reports or clinician administered interviews) (161). A meta-analysis of 61 studies using rTMS sham-controls for adult depression found a placebo effect size of 0.8 with the magnitude of the effect positively correlated with year of publication (162). Increasing placebo response over time may be an outgrowth of public/media attention, technologically more sophisticated devices (such as neuronavigation), enrollment of patients who are not “treatment resistant” (163, 164), and improved disguising of sham coils (165). The RCT in adolescents resulted in a sham effect size of 1.15 and a response rate of 36.4% (not significantly lower than the active rTMS effect size of 1.27 and response rate of 41.7%) (7). This higher placebo response rate in adolescents is also present in drug trials for adolescent depression (166) where placebo responses rates range from 24 to 60% (167–174).

To determine the efficacy of rTMS for adolescent depression, future RCTs will have to address the higher placebo effect rate. Aside from the conventional “active vs. sham” design alternative approaches such as inclusion of a placebo run-in, giving an active comparator, or applying statistical analysis techniques such as Growth Mixture Modeling (GMM) to capture unobserved subject heterogeneity in trajectories may be considered. The last approach requires dense collection of outcome measures over the course of rTMS and during follow-up. GMM was used to analyze data from an antidepressant trial; the placebo response trajectory deviated from the two active drug response trajectories (175). This technique has also been used to evaluate whether biomarkers, such as quantitative electroencephalography (qEEG), can predict antidepressant response (176).

Beyond the placebo response rate, other features in the study design for the RCT may have contributed to the failed outcome, including: the choice of the “5-cm rule” targeting strategy, broad inclusion criteria, within group variability in response, and insufficient sample size. The rTMS protocol, targeting strategy, frequency of stimulation, and dosage influence effect size in rTMS trials. Optimizing these factors in a neurodevelopmentally informed way could therefore increase effect sizes.

## Limitations of the Existing Literature and Proposed Resolutions

### Impact of Stimulation Parameters on Effect Size

The majority of clinical trials for both adult and adolescent depression have applied 10 Hz left hemisphere DLPFC rTMS. However, a meta-analysis of rTMS RCTs, encompassing over 4,000 patients and 81 trials, concluded that sequential bilateral stimulation to the right (1 Hz) and left (10 Hz) DLPFC was the most effective method (177). Only two adolescent depression trials used bilateral stimulation. Though safety and efficacy are the primary goals of novel treatment development, feasibility and tolerability also need to be considered. The standard 10 Hz protocol is burdensome for the patient and provider; daily sessions require over half an hour (up to an hour for bilateral stimulation protocols) and at least 6 weeks of treatment. Use of iTBS protocols can reduce this burden with a single session of iTBS taking only 3 min, allowing for accelerated protocols and shorter treatment courses (62).

iTBS protocols were designed to closely mimic endogenous theta/gamma rhythms of the brain and induce long-term potentiation-like (LTP-like) plasticity. Though early iTBS studies targeted primary motor cortex, this protocol has also been applied to other brain regions including the DLPFC. iTBS over the DLPFC has been shown to induce long-term changes in local cortical excitability (178, 179), reduce GABA and glutamate/glutamine levels, and alter network connectivity (180). On a behavioral level, iTBS led to improved performance on the ability to inhibit automatic responses and working memory tasks in a small study of healthy volunteers (181). In a non-inferiority study iTBS and 10 Hz rTMS both reduced symptoms of depression in adults with similar safety, tolerability and efficacy (182). A meta-analysis of adult studies showed a response rate of iTBS of 35.6% (42/118) vs. 17.5% (18/103) with sham, a pooled odds ratio of response and remission of 2.7 and 1.9, respectively, and an effect size of 1.0 (183). Though promising in adults, iTBS has not been evaluated in an RCT in adolescents and faces the challenge of the aforementioned higher sham response rates. However, the mechanism of action of iTBS may be particularly well-suited to the intrinsic neuroplasticity of the adolescent brain. Furthermore, the ability of iTBS to modulate aberrant neurotransmitter function and pathological connectivity is well-matched to the reported DLPFC pathophysiology of adolescent depression.

Different rTMS targeting strategies yield differences in precision, reliability, and effect size. In six of 12 studies (Table 2) of adolescent depression the targeting strategy was scalp-based, four used MRI-guidance, and two provide no data. Scalp-based targeting is unreliable and imprecise for localizing DLPFC (184–188). MRI-based neuronavigation used for rTMS shows larger effect sizes (127, 130, 131). For treatment of depression, one study in 51 depressed adults found a moderately larger effect for MRI-based neuronavigation than standard targeting (Hedges g_s_ = 0.64) (127). Even when using MRI-based neuronavigation, there are uncertainties about the optimal target for treating depression. Most studies target DLPFC, a large cortical area that is functionally connected to the Frontoparietal Network (also known as the Central Executive Network), the Default Mode Network, and the Salience Network (93, 189). All of these networks may be affected in depression and could be influenced by DLPFC stimulation. Using a standard “figure of 8 coil,” a shift of as little as 0.5–1 cm can differentially affect one or more of these networks (190). Furthermore, there is a large variation in individual functional brain circuitry (191). Thus, especially during adolescence when these networks are in flux, using patient-specific functional neuronavigated rTMS to precisely target and modulate one or more of these networks, could lead to larger effect sizes (192).

Larger effect sizes are also seen in protocols that apply more sessions and more pulses *per session* (193–195). A meta-analysis of number of sessions and pulses/session to treat depression found the average effect size increased from 0.43 to 2.74 when the number of sessions increased from 5 to 20; the maximum mean effect size (5.47) was seen with 20 or more sessions and more than 1,200 pulses/session (193). However, only five of the 12 studies of adolescent depression applied 20 or more sessions. Increasing the number of sessions *per day* above the convention of one session daily would increase efficiency. The conventional procedure results in a standard course of treatment of 6 weeks or more. Accelerated protocols with 2–10 session per day applying the same number of total sessions appear to show equivalent safety and efficacy (157, 195–199) with the pace of improvement showing a direct relationship with the cumulative number of sessions. Additionally, neuroimaging studies find that, like standard protocols, accelerated protocols result in changes on neurochemical and functional connectivity biomarkers of depression (103, 200). When applying more than one session daily, intersession interval influences additive effects. Basic research studies on LTP find that the level of LTP is doubled when a second TBS train is applied after 60 min, but if applied after only 10, 30, or 40 min there was no cumulative effect (201). That being said, neurodevelopmental factors could affect optimal intersession intervals and has yet to be determined. Piecing these protocol parameters together, a proposal for the most favorable balance of feasibility, efficacy and efficiency is up to five sessions/day, a 60-min intersession interval, and at least 20 total sessions (202). Such a treatment course can be completed in 1 week (instead of 6 weeks for once daily treatments). The value of swift antidepressant interventions becomes particularly relevant during the COVID pandemic, when access to therapy can be hindered and also adherence to longer therapy is a greater problem (203).

### Within-Group Variability: Impact of Inter-individual Heterogeneity and Intra-individual Brain State on Effect Size

Clinical heterogeneity may contribute to reduced therapeutic response in studies of adolescents (204, 205). Adolescent patients present with a diverse range of symptoms (205), clinical courses (206), and responses to treatment (16, 204, 207, 208). We and others have thus suggested that underlying pathophysiology may account for the observed clinical heterogeneity (209). Given this heterogeneity, one would not expect to find a single “one size fits all” optimal treatment for adolescent depression (210). To address clinical heterogeneity, one could increase the sample size, allowing for subgroup analyses, or focus on a narrower phenotype.

Attempts have been made to define clinical subtypes and brain-network-based biotypes, but these have been difficult to replicate (211). One promising symptom domain for targeted treatment is anhedonia/dysphoria. Anhedonic/dysphoric symptoms seem to be reliably associated with PFC-cingulate network dysfunction (212–215) and have been shown to be particularly responsive to neuronavigated rTMS to the DLPFC (100). This raises the possibility that reducing sample heterogeneity by enrolling with primarily anhedonic/dysphoric symptoms might increase power to observe an effect. Such an approach was successfully used in a recent pharmacological trial (216).

A different approach is to focus treatment on suicidal ideation, the most serious risk to patient safety. The literature on efficacy of rTMS for suicidality paints a mixed picture. A pooled analysis of 19 depressed adolescents who received open-label rTMS showed decreased suicidal ideation over the course of treatment (153). This decrease in suicidality, corresponded to an overall decrease in severity of depression symptoms (153). A retrospective analysis of 332 depressed adult patients who received rTMS also reported improvements in suicidality (217) and a review (*n* = 593) and naturalistic study (*n* = 43) of rTMS in adult MDD found consistent improvements in depressive symptoms and suicidality in open-label trials. These positive findings are contrasted with results from sham-controlled trials that have failed to show significant group differences in the reduction of suicidal ideation (210). While it is possible that, compared to conventional pharmacotherapy, rTMS might be a safe, faster means of reducing suicidal ideation; larger, sham-controlled RCTs employing the most current sophisticated methods will be needed to conclusively demonstrate efficacy. Furthermore, the immediate therapeutic effects of ketamine may soon become the standard for speed of reducing suicidality against which all other interventions must be compared.

For both safety and feasibility, most adult and adolescent rTMS studies have allowed participants to continue their current medications (218). Safety reviews suggest that rTMS in those receiving stable doses of antidepressant medication does not increase the risk of adverse events (82); however, this increases within-group variability in neurochemical state and decreases statistical power. To control for variability in neurochemical state, investigators could enroll only participants who have been withdrawn from all psychotropic medications; however, withdrawing symptomatic patients from their medications introduces safety concerns of increased suicidal ideation and withdrawal-related side-effects. It demands close medical monitoring. A more feasible approach would be to require participants to maintain a steady medication dose and to apply a within-subject model controlling for baseline severity as the primary outcome measure. While this would not eliminate variability across participants, it reduces the effects of neurochemical brain state on the primary outcome measure. Combining rTMS and pharmacological treatment is another novel multimodal intervention being developed in adults that could be extended to adolescents (219). Notably, recent data suggest that combining antidepressant medication with a course of rTMS may in fact have a greater benefit in adolescents than in adults (150).

In order to increase tolerability of the treatment, adolescent studies have allowed patients to read, watch TV, or listen to music during rTMS sessions. The difference in behavioral engagement/arousal this causes is another potential source of within-group variability. While these distraction strategies are common in everyday practice with rTMS, they are a concern for treatment trials. Factors such as attention, arousal and mood state have been shown to affect modulation of excitability by rTMS (220–223). Thus, if the adolescent is even passively engaged in an unrelated activity, this may impact the effect of the rTMS. Applying shorter stimulation protocols (e.g., iTBS) may reduce the need for co-occurring activities to make the session more tolerable. Alternatively, one can transiently modify the patient's cognitive state by presenting stimuli or engage them in a task that engages the same brain networks as the rTMS target. In this way, rTMS can be combined with the behavioral task in order to amplify the impact on the targeted network (76). Studies of rTMS for post-traumatic stress disorder (PTSD), smoking cessation, and OCD have shown increased treatment response when the participant's symptoms were provoked [e.g., by asking questions about thoughts, images, or impulses related to their obsessions or compulsions or asking the patient to perform a task related to their symptoms (224)] immediately prior to rTMS stimulation (225–227). One could also consider pairing stimulation with concurrent behavioral interventions such as CBT (228).

### The Importance of Imaging Biomarkers in rTMS Treatment Development

Given the significant time-investment necessary for rTMS treatment, many have sought to identify early predictors of later response. A recent retrospective study of 101 patients who received 4 weeks of rTMS treatment found that a lack of clinical response at the midway point predicted non-response with 88% accuracy (229). However, this still requires the patient to undergo 10 treatment sessions. Identifying intrinsic neurophysiological signatures or measures that can be obtained after one or two rTMS sessions would be preferable (229). Brain imaging techniques are increasingly capable of obtaining measures of brain function at cellular/molecular and network levels (96, 97, 230, 231). MRS can yield measures of GABA and glutamate neurotransmitter functioning (230, 231). BOLD fMRI and RSFC (96, 97) have been proposed as tools for neuroimaging biomarkers. RSFC as measured by fMRI is stable across development (232, 233) and reproducible (234) at a group level. RSFC in adolescent depression has been shown to differentiate (at a group-, not at the individual-level) symptom severity (235), symptom domains (213, 236, 237), onset (238), and course of disease (239, 240). Furthermore, RSFC in fronto-parietal, cingulo-opercular/ventral attention, and default mode networks has been used to predict individual differences in adolescent brain maturity and executive functioning (241). Within individuals, however, most commonly used sequences are too short to produce reliable RSFC measures (242), but with longer sequences (243, 244) or novel sequence types (244) it may be a reliable measure. rTMS trials in adolescent depression would benefit from dense collection of reliable neuroimaging measures at baseline, post-treatment, and follow-up. At baseline, neuroimaging measures may allow investigators to customize treatment to the unique neurodevelopmental state of each adolescent's developing cortex. Though group data appear promising, the benefit of using RSFC profiles for patient-specific treatment decisions is currently only theoretical.

Neuroimaging measures can also serve as predictive biomarkers for response to rTMS. Multiple studies have linked intrinsic functional connectivity between the targeted region of PFC and ACC, anterior insula and striatum with later response to rTMS (74, 125, 200, 245–254) and other antidepressant treatments (101, 255–258). Interleaved rTMS/fMRI has shown acute effects on BOLD activation of both local and distal brain regions immediately following 1 Hz DLPFC rTMS in adults with MDD (89). Though research is promising in this area, replication and prospective examination of these predictive biomarkers will be critical prior to clinical implementation.

To facilitate clinical translation of such biomarkers, the National Institute of Mental Health (NIMH) has introduced “fast-fail” trials. This initiative employs an experimental therapeutics platform to quickly identify devices, protocols or compounds that should be considered for more extensive testing (259). These types of trials may be especially useful in adolescent depression where there is a clear urgent public health need for novel therapeutics, but still a great deal of uncertainty in the neural targets and biomarkers that reliably predict treatment response. The “fast-fail” initiative has recently led researchers to report successful target-engagement of a novel kappa-opioid receptor antagonist for the treatment of anhedonia in adult MDD (216). Thus, extending this approach to adolescents is both timely and feasible.

## Conclusions

Treating adolescent depression is fraught with the challenges of heterogeneity of the clinical phenotype, the high placebo response rate, and the breadth of neurodevelopmental changes during puberty. While the efficacy of rTMS for adults with treatment resistant MDD is supported by multiple, adequately powered RCTs, evidence in adolescent depression is scanty and has many limitations. The safety profile of rTMS for adolescent depression appears to be desirable but many parameters, including the most favorable approaches, have yet to be determined.

rTMS is worthy of further investigation for this vulnerable population. We propose that future adolescent depression rTMS trials: (1) be designed with 20 or more sessions (2) select a narrow clinical phenotype (e.g., select for anhedonia); (3) test for the possibility of individualization by including neuroimaging biomarkers (e.g., RSFC); (4) employ an experimental therapeutics approach (5) allow for robust inferences by being adequately controlled and powered with realistic effect size estimates. Such trials, if successful, may establish paradigms for larger, pivotal trials that clarify whether rTMS is an effective treatment for adolescent depression. Furthermore, they are likely to advance our understanding of the pathophysiology of this disorder.

## Author Contributions

LO drafted and revised the manuscript. MH conducted the literature review under the supervision of LO. DN, KT, SL, and AS contributed to the drafting and revision of the manuscript. All authors approved the final submitted manuscript.

## Conflict of Interest

The authors declare that the research was conducted in the absence of any commercial or financial relationships that could be construed as a potential conflict of interest.
